# Social Attention in Electronic Picture Books with Social Scenes for Children with Autism Spectrum Disorder: Insights from Eye-Tracking Studies

**DOI:** 10.3390/bs16040536

**Published:** 2026-04-02

**Authors:** Lintao Yang, Yan Chen, Meifen Chen, Xiaoqun Wang, Leyuan Liu

**Affiliations:** 1 College of Physical Science and Technology, Central China Normal University, Wuhan 430079, China; ltaoyang@ccnu.edu.cn (L.Y.); cyan2020112198@mails.ccnu.edu.cn (Y.C.); 2 School of Digital Media, Shenzhen Polytechnic University, Shenzhen 518055, China; chenmeifen@szpu.edu.cn; 3 School of Education and Psychology, Minnan Normal University, Zhangzhou 363000, China; wxq0894@mnnu.edu.cn; 4 National Engineering Research Center for E-Learning, Central China Normal University, Wuhan 430079, China

**Keywords:** autism spectrum disorder, electronic picture book, social attention, social scenes, eye-tracking

## Abstract

Electronic picture book free viewing can promote language comprehension ability and social cognitive abilities in children with autism by providing structured visual information. Understanding autism spectrum disorder (ASD) children’s visual attention patterns during electronic picture book free viewing can inform targeted educational research. The attentional preference of children with ASD toward electronic picture books with social scenes remains under-explored. This study aimed to understand the social attention of children with ASD during free viewing of electronic picture books with social scenes. Eye-tracking technology was used to record the visual behavior of 24 children with ASD viewing electronic picture books independently, and 25 typically developing (TD) children were selected as the control group. The results showed that children with ASD allocated less fixation time to social information in electronic picture books than TD children, with a clear difference in the fixation time spent on facial regions. Children with ASD neither displayed the same attention to happy facial expressions in electronic picture books as TD children nor did they show significant differences in attention to different emotions. These findings contribute to our understanding of visual attention patterns in children with ASD during electronic picture book free viewing and provide empirical evidence for future research on optimizing visual viewing guidance for children with ASD.

## 1. Introduction

Autism spectrum disorder (ASD) is a neurodevelopmental condition characterized by heterogeneous social and communicative differences, as well as restricted and repetitive behaviors and interests, which is widely associated with atypical patterns of social attention (American Psychiatric Association [APA], 2022; Di Giorgio et al., 2021). These characteristics are often associated with differences in social engagement, verbal and nonverbal communication, and adaptive behaviors. Differences in social communication affect social interactions between children with ASD and peers or teachers in educational settings (Wolstencroft et al., 2018). Approximately 30% of children with ASD maintain minimally verbal communication (i.e., speak no words, few words, or short sentences).

Electronic picture books represent a common digital format for presenting social scenes and text (Bai et al., 2022), which has been linked to variations in children’s cognitive, emotional, and social development (Sandgreen et al., 2021). As many children with ASD show a strong preference for digital media (Torun Yeterge, 2025; Valencia et al., 2021), electronic picture books provide a suitable context for examining their spontaneous social attention (Thompson et al., 2019).

Despite the potential benefits of electronic picture books for children with ASD, research examining social attention within this specific context remains limited. In contrast, social attention in children with ASD has been studied extensively using traditional image-based or video-based stimuli, often under instructed viewing conditions (Congiu et al., 2024). Social attention refers to an explicit attentional bias to focus and look at others, as well as where the attention is directed (Falck Ytter et al., 2023). At present, studies on social attention using images (Frost-Karlsson et al., 2019) or videos (Li et al., 2020; Özdemir et al., 2022) of natural scenes as stimuli have shown that children with ASD have different social attention than children with TD and have a reduced predisposition to social stimuli. For example, a recent study using videos of social play found that children with ASD showed reduced attention to social stimuli and spent less time looking at faces relative to TD children (Robain et al., 2021). However, Latreche et al. (2021) reported that the social orientation of children with ASD was not universally impaired, and the decline in attention to faces was not generalized across all contexts. It remains unclear whether such context-dependent differences in social attention extend to spontaneous unguided viewing of social-themed electronic picture books, a context that has received little attention in existing eye-tracking research.

The visual exploration of social scenes in children with ASD is associated with facial emotions (Bochet et al., 2021; Hutto et al., 2018). Reduced attention to faces in social scenes may lead to impaired emotional processing in ASD (Chevallier et al., 2012; Wieckowski & White, 2020). Current research has shown that individuals with ASD have defects in emotional processing (Masoomi et al., 2025; Styliadis et al., 2021; Van der Donck et al., 2021), such as an impaired ability to detect happy facial expressions (Sato et al., 2017). Although a study examining visual preferences for social versus nonsocial images suggested that children with ASD show an emotional preference for happy faces (Vacas et al., 2021), they may often miss nonverbal gestures and social cues, such as facial expressions, that help understand key nonverbal behaviors associated with electronic picture books (Chen et al., 2016). Accordingly, children with ASD do not necessarily show a preference for gazing at happy faces in unguided viewing of electronic picture books. In addition, it has been shown that individuals with ASD exhibit non-differential emotional responses to different facial emotions (Deschamps et al., 2015; Yeung, 2022). Therefore, further exploration of attention to the facial emotions exhibited by children with ASD may provide insight into details of their viewing of electronic picture books with social scenes.

Eye-tracking techniques are widely used to characterize the social attention of children with ASD (Bacon et al., 2020; Camero et al., 2021), and the technology has also been validated as a reliable tool for investigating attentional deficits in other neurodevelopmental disorders such as ADHD (Andreou & Argatzopoulou, 2025). The application of eye-tracking technology in viewing tasks can provide objective insights (Saputro et al., 2023), especially for visual pattern analysis of picture content (Alemdag & Cagiltay, 2018). In viewing electronic picture books with social scenes, children with ASD can build their understanding of pictures from social information and facial emotions, and each visual behavior may reflect their viewing characteristics in viewing electronic picture books. To gain a clear understanding of the details of the viewing process of children with ASD during unguided and spontaneous viewing, this study uses eye-tracking technology to track and record the visual behaviors of children with ASD while viewing electronic picture books and analyzes the social attention of children with ASD in these viewing materials. Based on previous studies and the design of this study, the following three hypotheses are proposed in this study.

**Hypothesis 1.**  *Children with ASD will show less attention toward social information in social-themed electronic picture books than TD children.*

**Hypothesis 2.**  *Children with ASD will allocate less attention to faces compared to TD children during electronic picture book viewing.*

**Hypothesis 3 (exploratory).**  *Children with ASD will show similar levels of attention across different facial emotions in electronic picture books.*

## 2. Materials and Methods

### 2.1. Participants

Children in the ASD group were recruited from an autism rehabilitation institution in Zhangzhou City. Inclusion criteria among children with ASD were as follows: a previous DSM-5-TR diagnosis of ASD made by a qualified clinical professional experienced in the assessment and diagnosis of ASD (American Psychiatric Association [APA], 2022). According to the Childhood Autism Rating Scale (CARS) for screening, the test scores were higher than 30 points. The study was approved by the Ethics Committee of Central China Normal University (Approval No. CCNU-IRB- 202305008b), and the parents and institutions were informed of the relevant details before starting the experiment to ensure their informed consent. Parents filled out the Autism Behavior Checklist (ABC), and all of the scores were above 53 points. Children with severe intellectual disability, epilepsy, or other major psychiatric disorders were excluded. The CARS and ABC scores were used solely for group characterization and not included as covariates in the main analysis.

The control group consisted of TD children recruited from elementary schools in the same geographical area as the children with ASD. TD children with any developmental disability (e.g., intellectual disability) or having any immediate family member with a diagnosis of ASD were excluded. Younger children with ASD have speech and comprehension dysfunction. It is difficult to perform intelligence tests; therefore, the intelligence of the two groups of children was not matched. Nevertheless, both groups were able to complete the picture-viewing task. None of the participants in either group had any visual impairments that could not be corrected with prescription lenses.

Fifty-five children participated in the study, including 30 with ASD and 25 with TD. Among them, six children with ASD were unable to complete the experiment because of crying and other behaviors. The final sample of 49 children who completed the experiment consisted of 24 children with ASD (20 boys, 4 girls; mean age = 4.7 years) and 25 controls (7 boys, 18 girls; mean age = 4.1 years). There was no significant difference in age (*t* (47) = −1.645, *p* = 0.107), but the sex ratio (*p* < 0.001) differed between the ASD and TD groups. Confounding by gender was avoided in the data analysis. Further examination of the data is presented in Table 1.

### 2.2. Apparatus

The electronic picture book was viewed on a computer set at the height of 48.7 cm. Under the normal display conditions of the iPad, the automatic playback function of the electronic picture book was used to record the screen as video material. The resolution of the video presentation screen was 1680 × 1050 pixels. The video of the electronic picture book was uploaded to the SMI RED 500 supporting software (Experiment Center). It was used to display the video to the participants, record the eye-gaze behavior captured by the eye-tracking unit (described below), and interpret the eye-gaze results. The SMI RED 500 remote eye-tracking unit and its software iView X software: Version 2.8 and Experiment Center were used to present the video material and record all participant data on each page of the electronic picture book. The eye-tracking unit was controlled by a laptop computer and did not require any equipment to be attached to the participant. The evaluation accuracy of the eye-tracking system was at the 0.5 level, the sampling rate was 120 Hz, and the coordinate data of the eyes were recorded separately. With the help of a small bouncing blob on the screen, the eyes were calibrated, and at least 100 ms of sustained gaze within a 1° angle of view was required to determine gaze.

### 2.3. Stimuli

**Figure 1:**
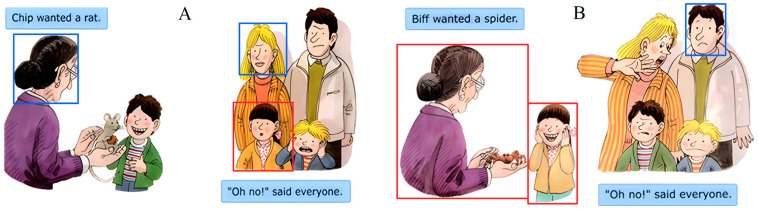
Part of the electronic picture book with social scenes and definitions of areas of interest. (**A**,**B**) show two pages with consecutive playback. Blue boxes denote facial and emotional regions (head region: top of head to chin), and red boxes denote social regions (full character: face and body).

The current study intended to observe children’s visual attention to the social information and facial emotions in electronic picture books. We chose an electronic picture book that contained social scenes, with fixed protagonists and a coherent storyline, since the experiment needed to avoid effects caused by different page sizes. This electronic picture book (as shown in Figure 1) is titled “The Pet Shop” and it describes the story of the three little protagonists (Biff, Chip, and Kipper) and their parents going to the pet store together. The social scenes on each page are in the pet shop, and the social information in the scene is that of the five protagonists. As different animals (e.g., mice, crabs, and snakes) appear in the scene, these protagonists show six basic emotions (anger, fear, disgust, happiness, surprise, and sadness). This particular book was chosen because it is suitable for children aged 4–7 years who are at the preliminary reading stage, and the text is short and simple to comprehend. This book is eight pages long and was obtained through the Oxford Reading Tree. The Oxford Reading Tree is a popular online reading scheme for children that is used in various countries worldwide. Specifically, pages 2, 3, and 4 were selected as the three-page segment for the formal experiment, as they include coherent content, clear social interactions, and distinct facial emotional expressions.

A short line of text (4–8 words) on each page was located at the bottom or top fifth of the page, and the social scenes were in the middle. Previous researchers have created three conditions commonly used in electronic picture books with social scenes: (a) the sound presentation of electronic picture books, (b) the brief background of social scenes in electronic picture books, and (c) clear emotional expressions in electronic picture books. Children viewed the electronic picture book only once, and these three conditions were consistently applied across all three pages. Each page was shown to participants for a fixed time (31–33 s, *M* = 32 s, *SD* = 0.82 s), and the total presentation time of the electronic picture book was 96 s.

### 2.4. Procedures

**Figure 2:**
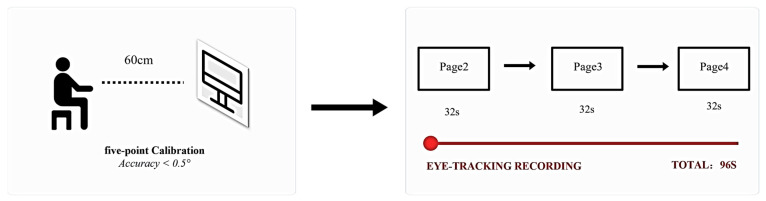
Schematic of the experimental procedure. (**Left**): Five-point eye-tracking calibration (accuracy < 0.5°) with participants seated 60 cm from the monitor. (**Right**): Three consecutive e-book pages (Pages 2–4) were presented at 32 s per page (totalling 96 s). Eye-tracking data were recorded continuously throughout all pages.

Before starting the test, all participants and their guardians received a thorough explanation of the procedure and apparatus and written informed consent was obtained from all participants and their parents. The sessions in which the children viewed the electronic picture book took place at school in a quiet and bright environment that was not being used for other activities. Before presenting the electronic picture book, each participant completed and passed the standard five-point calibration procedure. Following the calibration, the children sat on chairs placed 60 cm away from the eye-tracker screen, or in the lap of a parent. The children were asked to look freely at the electronic picture book displayed on the monitor and listen to the narration by the computer voice. No additional task instructions or viewing guidance were provided, ensuring that visual exploration remained unguided and spontaneous (Backhaus et al., 2020). A schematic overview of the experimental procedure, including calibration and stimulus presentation timing, is provided in Figure 2.

### 2.5. Data and Statistical Analysis

To quantify the visual gaze pattern, the regions of interest (i.e., the social, facial, emotional, and effective viewing regions) were selected for further analysis. Each social region contained social information that simultaneously included both the face and body of the protagonist. There were five social regions corresponding to the five main protagonists (Biff, Chip, Kipper, Mother, and Father). The facial regions included the region from the leftmost corner of the head to the rightmost corner of the head in the horizontal direction and the region from the top of the head to the bottom of the chin in the vertical direction for each social region. As the facial emotions in this electronic picture book were clearly expressed, the emotional regions in this study were like the method used to determine facial regions. Owing to the difference between the size of the electronic picture book and the screen, there were other black regions without content, in addition to the stimulus presented by the stimulus presentation monitor. In this study, the effective viewing regions were defined as electronic picture books that did not contain black regions. These definitions of areas of interest are illustrated in Figure 1.

In the present study, using BeGaze Version 3.7, a built-in data analysis software, all fixation data were abstracted. Two eye-tracking parameters were analyzed. The first parameter was fixation time (%; FT), the ratio of the total region of interest gaze time to total stimulus time, reflecting the absolute attention displayed by the participants in a certain region of interest (Holmqvist et al., 2011). The second parameter was the first fixation duration (ms; FFD), defined as the fixation duration of the first entry into the region of interest for more than 100 ms, reflecting the duration of time the participant’s eyes were drawn to the regions of interest view for the first time or the time that might have been spent in the thinking process (Staub & Benatar, 2013).

As there were significant differences between groups in terms of sex, it was considered in subsequent analyses. This study used a controlled design with a two-way analysis of covariance (ANCOVA) (groups × emotions) to compare two eye-tracking parameters in children with ASD and TD children, and sex was controlled as a covariate. As multiple observations were obtained from each participant across emotional conditions and regions of interest, the potential dependencies in the data are acknowledged, and results are interpreted with caution. All statistical analyses were conducted using IBM SPSS Statistics 27 and JASP version 0.14. An alpha level of *p* = 0.05 was used to determine if there were significant results. Due to the exploratory nature of this study, no a priori or post hoc power analysis was performed. The effect sizes (partial eta-squared, for F statistic) were reported together with the *p*-value of the significant main effect, where *η*^2^ values higher than 0.01, 0.06, and 0.14 are generally considered to reflect small, medium, and large effects, respectively. Small effect sizes are reported to show the practical magnitude of group differences, rather than only interpreting statistical significance. While all the significant main effects were reported, we focused on significant interactions with groups because of the large number of possible interactions and our focus on group differences. If a significant interaction effect was found, simple main effects were further examined.

## 3. Results

### 3.1. The Overall Electronic Picture Book

**Figure 3:**
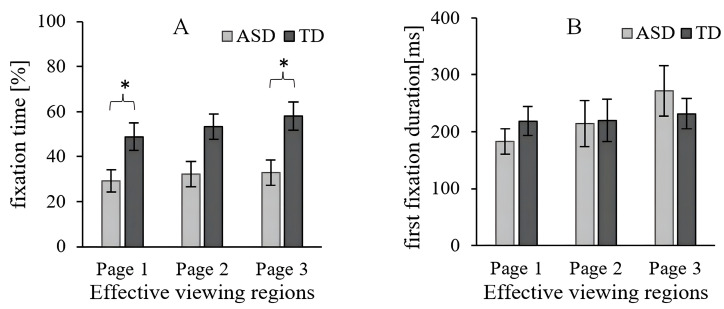
Eye-tracking data for the effective viewing regions. (**A**) Fixation time (%; FT). (**B**) First fixation duration (ms; FFD). Level of significance: * *p* < 0.05.

To determine whether the groups differed on the three-page electronic picture book, a two-way ANCOVA (Group × Page) was conducted on FT and FFD. A significant main effect was found in the two groups [*F*(1, 139) = 16.225, *p* < 0.001, *η*^2^ = 0.103] in the statistical analysis of FT; however, no differences were found among the different pages [*F*(2, 139) = 0.622, *p* = 0.538, *η*^2^ = 0.008] and effect interaction [*F*(2, 139) = 0.126, *p* = 0.882, *η*^2^ = 0.002]. Figure 3A shows the total number of FT spent in the active viewing region when viewing the entire screen for the ASD group and the TD group.

No significant effects were found for the two groups [*F*(1, 139) = 0.006, *p* = 0.940, *η*^2^ = 3.942 × 10^−5^], different pages [*F*(2, 139) = 1.191, *p* = 0.307, *η*^2^ = 0.017], and effect interaction [*F*(2, 139) = 0.641, *p* = 0.529, *η*^2^ = 0.009] for FFD. These results indicated that children with ASD and TD pay similar attention to each page of the electronic picture book when watching the experimental scenes (as shown in Figure 3B).

Due to the centrality of protagonists in the content of children’s scene-based electronic picture books, we focused on analyzing the role of eye-gaze patterns in the relevant characteristics of different protagonists. To prevent the possible effect of gender, the gender of the protagonists in the scene was selected for statistical analysis. The two-way ANCOVA (group × protagonist’s gender) on FT found that the interaction effect [*F*(1, 1464) = 3.655, *p* = 0.056, *η*^2^ = 0.002] was not significantly different. Similar results were also found in the statistical analysis of FFD (interaction effect [*F*(1, 1464) = 0.142, *p* = 0.706, *η*^2^ = 9.581 × 10^−5^]). Therefore, there was no significant difference in the visual characteristics between the two groups of protagonists of different genders in the electronic picture book.

### 3.2. Distribution of Attention in Social Information

When we divided the social regions in the electronic picture book, the two-way ANCOVA (Group × Social) on FT yielded a significant but small overall main effect for the two groups [*F*(1, 1458) = 24.147, *p* < 0.001, *η*^2^ = 0.016] and an interaction effect [*F*(4, 1458) = 3.272, *p* = 0.011, *η*^2^ = 0.009], but there was no significant difference for the five social regions [*F*(4, 1458) = 1.982, *p* = 0.095, *η*^2^ = 0.005]. To explore these effects, a simple main effect analysis was conducted using the social regions as a moderator factor. Although FT did not differ between groups for Father [*p* = 0.970], it was higher in the TD group than in the ASD group in other social regions (Biff [*p* = 0.004], Chip [*p* = 0.042], Kipper [*p* = 0.002], and Mother [*p* = 0.003]).

**Figure 4:**
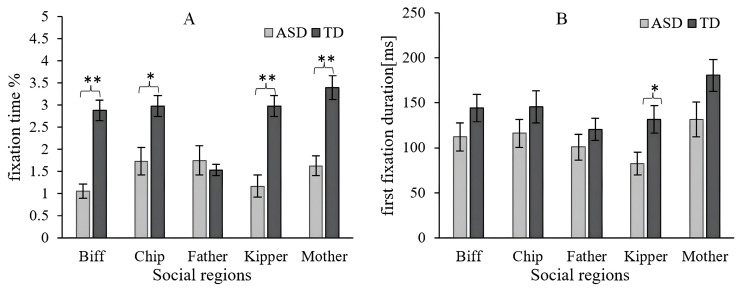
Eye-tracking data for the social regions. (**A**) Fixation time (%; FT). (**B**) First fixation duration (ms; FFD). Level of significance: * *p* < 0.05, ** *p* < 0.01.

The two-way ANCOVA (Group × Social) on FFD indicated the main effects of group [*F*(1, 1459) = 8.555, *p* = 0.003, *η*^2^ = 0.006] and social region [*F*(4, 1459) = 3.083, *p* = 0.015, *η*^2^ = 0.008], but there was no significant difference in effect interaction [*F*(4, 1459) = 0.335, *p* = 0.855, *η*^2^ = 9.041 × 10^−4^]. Hence, there was no difference in FFD between the social regions in the ASD and control groups. The TD group displayed a tendency to look more at the social regions than the ASD group in each role. As shown in Figure 4A, the FT on the social regions in the ASD group (*M* = 1.468, *SD* = 3.148) was significantly lower than for the TD group (*M* = 2.753, *SD* = 4.909), and similar results were found in Figure 4B for FFD (ASD group: *M* = 108.723, *SD* = 187.411; TD group: *M* = 144.543, *SD* = 194.986).

**Figure 5:**
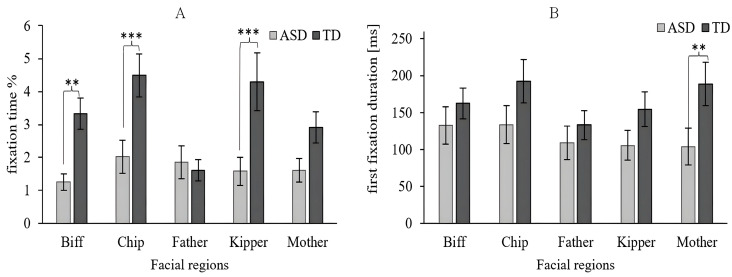
Eye-tracking data for the facial regions. (**A**) Fixation time (%; FT). (**B**) First fixation duration (ms; FFD). Level of significance: ** *p* < 0.01, *** *p* < 0.001.

This study further analyzed attentional differences in face regions in social scenarios. The two-way ANCOVA (Group × Facial) for FT in the facial region indicated the main effects of group [*F*(1, 723) = 16.845, *p* < 0.001, *η*^2^ = 0.022] and facial region [*F*(4, 723) = 2.728, *p* = 0.028, *η*^2^ = 0.014]. The Group × Facial interaction [*F*(4, 723) = 2.662, *p* = 0.032, *η*^2^ = 0.014] was significant. A simple main effect analysis was performed on the FT to explore these possible effects, using the group as a simple effect factor and facial region as a moderator factor. As shown in Figure 5A, there were significant group differences, as reflected for Biff [*p* = 0.028], Chip [*p* = 0.003], and Kipper [*p* = 0.002].

However, the two-way ANCOVA (Group × Facial) for FFD in the facial region yielded an overall main effect for the groups [*F*(1, 723) = 5.062, *p* = 0.025, *η*^2^ = 0.007] and for social regions [*F*(4, 723) = 0.893, *p* = 0.468, *η*^2^ = 0.005]. As shown in Figure 5B, there was no significant interaction effect on the two-way interaction between group and facial region [*F*(4, 723) = 0.492, *p* = 0.742, *η*^2^ = 0.003].

### 3.3. Distribution of Attention in Facial Emotional

**Figure 6:**
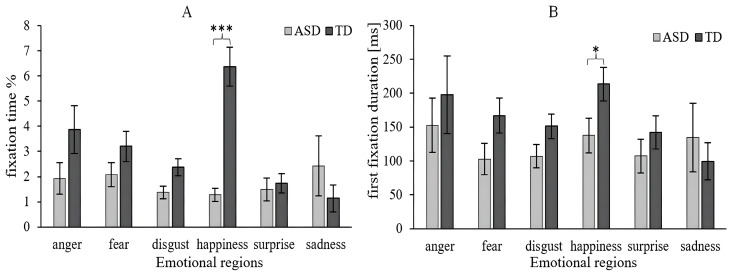
Eye-tracking data for the emotional regions. (**A**) Fixation time (%; FT). (**B**) First fixation duration (ms; FFD). Level of significance: * *p* < 0.05, *** *p* < 0.001.

The two-way ANCOVA (Group × Emotion) on FT yielded a significant but small main effect for groups [*F*(1, 721) = 9.226, *p* = 0.002, *η*^2^ = 0.012]. The FT in the ASD group (*M* = 1.664, *SD* = 3.536) was significantly less compared to the TD group (*M* = 3.329, *SD* = 5.213). The main effect of emotion [*F*(5, 721) = 4.767, *p* < 0.001, *η*^2^ = 0.030] was significant and had a significant effect on the interaction between emotion and the group [*F*(5, 721) = 6.598, *p* < 0.001, *η*^2^ = 0.042]. A simple main effect analysis was conducted with the group as a simple effect factor and emotion as a moderator factor for FT in each region of interest. As shown in Figure 6A, the simple main effect of the group was not significant for anger (*p* = 0.198), fear (*p* = 0.351), disgust (*p* = 0.087), surprise (*p* = 0.737), and sadness (*p* = 0.085), but was significant only for happiness (*p* < 0.001). Notably, children with ASD showed no significant differences in viewing time across the six emotion categories, indicating a global, undifferentiated pattern of attention rather than specific sensitivity to any emotion.

The two-way ANCOVA (Group × Emotion) on FFD indicated the main effects of the group [*F*(1, 721) = 2.207, *p* = 0.138, *η*^2^ = 0.003] and emotion [*F*(5, 721) = 1.468, *p* = 0.198, *η*^2^ = 0.010]. There was no significant difference in the interaction effect [F(5, 721) = 0.595, *p* = 0.704, *η*^2^ = 0.004]. As shown in Figure 6B, there was a significant difference in FT and FFD between the ASD and normal groups, only for happiness.

To test for differences in FT for different emotions among children with ASD, the one-way ANCOVA (Emotion) showed no significant difference toward different emotions [*F*(5, 352) = 0.851, *p* = 0.514, *η*^2^ = 0.012]. The FTs of children with ASD for each of the six emotions were as follows: anger [*M* = 1.933, *SD* = 3.093], fear [*M* = 2.074, *SD* = 4.649], disgust [*M* = 1.370, *SD* = 2.428], happiness [*M* = 1.282, *SD* = 2.199], surprise [*M* = 1.488, *SD* = 3.190], and sadness [*M* = 2.425, *SD* = 5.832]. The one-way ANCOVA (Emotion) on FFD also showed no significant difference toward different emotions [*F*(5, 352) = 0.507, *p* = 0.771, *η*^2^ = 0.007].

## 4. Discussion

The current study aimed to investigate the social attention of children with ASD while free viewing electronic picture books with social scenes. The central thesis of this study was to explore the visual attention of children with ASD to social information and facial emotions in electronic picture books with social scenes using eye-tracking techniques. Specifically, first fixation duration (FFD) indexes early attentional orienting, while fixation time (FT) reflects sustained attention and visual preference. TD children were included as the control group. The study revealed some interesting and important findings, discussed below from three perspectives.

### 4.1. Early Cognitive Processing and Sustained Attention

This study found that children with ASD did not show differences in FFD from TD children in the social, facial, and emotional regions. Children with ASD did not differ from TD children in the speed of early attentional orienting (Ridderinkhof et al., 2020). There was no difference between the groups in the duration of the first fixation, but some differences were reflected throughout the free viewing of the electronic picture book with social scenes. Although studies have demonstrated that picture books, by their own nature (text, images), have proven to be a suitable genre to engage children with ASD (Lian et al., 2022), without additional instruction, children with ASD may not spontaneously prioritize the main content in an electronic picture book. Therefore, future research may examine whether targeted guidance could support sustained attention during free viewing.

### 4.2. Visual Attention to Social Information in Electronic Picture Books

The total time children with ASD spent on social regions in electronic picture books with social scenes was less than TD children, demonstrating that children with ASD pay less attention to social information in electronic picture books than TD children do (supporting Hypothesis 1). Most eye-tracking studies have shown that individuals with ASD spend less time attending to social information than TD controls (Congiu et al., 2024; McLaughlin et al., 2021; Tang et al., 2022). Using videos of adults and infants playing together as stimuli, Shic et al. (2011) found that children with ASD pay less attention to the activities of others compared to TD children. However, Frost-Karlsson et al. (2019) used social scenes with or without a person present in the image as experimental stimuli and concluded that no significant differences were found between the two groups in terms of the total time spent focusing on social scenes. A meta-analysis suggested that these different results may be brought about by social content, with higher social content of the stimulus (i.e., more than one person) linked to reduced social attention in ASD (Chita-Tegmark, 2016). Therefore, the relatively low social attention of children with ASD in electronic picture books with social scenes of high social content may reflect distinct attentional patterns or visual exploration strategies when processing social information involving multiple individuals.

This study further explores the detailed attention to the face in the social regions. The results showed that children with ASD pay less attention to the facial regions in electronic picture books with social scenes than TD children (supporting Hypothesis 2). Several studies using pictures have found that participants with ASD and TD controls spent a similar proportion of time gazing at faces (Fletcher-Watson et al., 2009; Freeth et al., 2010). These studies used stimuli from only one individual. In contrast, when a set of stimuli included photos of at least two people interacting, individuals with ASD were found to look at faces less than TD controls (Sumner et al., 2018). In TD children, increasing the social content of stimuli (e.g., number of people) increases the amount of time spent looking at faces (Kaliukhovich et al., 2022). Thus, for children with ASD, viewing electronic picture books with multiple characters was associated with less gaze time toward facial regions compared to TD controls. These findings suggest that future research could explore whether instructional guidance or visual scaffolding may improve social attention in children with ASD.

### 4.3. Visual Attention to Facial Emotion in Electronic Picture Books

In our study, when emotional faces were in competitive conditions with other emotional faces, children with ASD were found to not show as much attention to happy emotions in electronic picture book scenes as TD children. In contrast, Vacas et al. (2021) found a typical preference for happy expressions in children with ASD. When six basic emotions were present simultaneously in the population, Farran et al. (2011) found that the identification of happy expressions was the fastest response and resulted in the fewest errors in detecting happiness, revealing a typical preference for happy expressions in individuals with ASD. In the previous discussion, the present study exploring visual preferences for electronic picture books with social scenes found that children with ASD pay less attention to social and facial regions. Some studies have revealed that reduced attention to faces is associated with differences in emotion recognition and expression in individuals with ASD (Wieckowski & White, 2020). Therefore, the results of the present study suggest, to some extent, that the reduced attention to social and facial regions in children with ASD accompanies lower attention to happy expressions in such contexts, though these findings do not directly address emotion recognition ability.

Children with ASD do not differ significantly in their visual attention to the six basic emotions in the electronic picture book with social scenes (supporting Hypothesis 3), indicating a non-selective pattern of attention toward emotional expressions rather than rather than an emotion-specific deficit. Previous research has noted distinct patterns in how individuals with ASD process others’ emotions, whether positive (e.g., happy) or negative (e.g., sad) (Liu et al., 2023; Sato et al., 2017). Children with ASD pay less attention to facial expressions that could help them understand social scenes, which may reflect a different prioritization of nonverbal social cues rather than a primary deficit in emotion processing. The absence of an emotional visual focus in the spontaneous free-viewing behavior of children with ASD in this study may suggest that emotional information is not prioritized in their attentional allocation, which may be influenced by task demands or lack of external guidance rather than attentional limitations.

### 4.4. Limitations

There are some limitations of this study that need to be noted, as well as some recommendations for future studies. First, the small number of children assessed in both groups limited our ability to compare the results with TD children. Future studies could replicate this study with larger samples. Second, the present findings are descriptive and do not allow causal inferences, and participant characteristics including cognitive and language levels were not fully described. Further comprehensive studies could adopt minimal experimental manipulations to support more causal interpretations for educational practice. Third, this study did not comprehensively consider personal factors, and the e-book was presented as a narrated video with fixed pacing, which may limit its ecological validity. Future studies could incorporate these factors and adopt more ecologically valid designs. As such, the present findings are specific to this unguided free-viewing task and may not directly generalize to other paradigms.

## 5. Conclusions

This study utilized an eye-tracking approach to examine social attention to social information and facial emotions of children with ASD while viewing electronic picture books with social scenes. We found that children with ASD allocated different attention to social information compared to TD children, and spent significantly different amounts of time gazing at faces. Regarding emotional visual attention, in cases where the face in the electronic picture book was presented simultaneously with other emotional faces, we did not observe a preference for happy expressions in children with ASD as is seen in TD children. Children with ASD did not show any significant differences between emotions during the viewing process. Alternatively, the present study found that there may be no group differences in early visual processing when viewing electronic picture books. The results of this study further our understanding of the visual attention of children with ASD in viewing electronic picture books, and raise testable hypotheses for future research on optimizing electronic picture book viewing and relevant educational practices for children with ASD.

## Figures and Tables

**Table 1:** Sample characteristics.

	ASD	TD	t-Value	*p*-Value
N	24	25		
Sex (boys/girls)	20/4	7/18		<0.001
Range of age (years)	3.1–9.0	3.0–6.0		
Mean age (±SD)	4.7 ± 1.55	4.1 ± 0.88	−1.645	0.107

## Data Availability

The datasets generated for this study are not publicly available due to ethical restrictions. The data supporting the findings of this study are available from the corresponding author upon reasonable request.
